# Serum adropin as a potential renoprotective factor in diabetic kidney disease: evidence from a Chinese elderly cohort and an experimental mouse model

**DOI:** 10.3389/fendo.2026.1893876

**Published:** 2026-07-13

**Authors:** Ruohan Jiang, Xianfa Xuan, Jing Ye, Xiulan Guo, Hanxiang Jiang, Siyu Zhang, Zhaoxia Ruan, Ting Wu, Jia Wang, Chen Tang, Yiqin Zhang, Jie Zhang

**Affiliations:** 1 Department of Nephrology, The Second Affiliated Hospital of Xiamen Medical College, Xiamen, Fujian, China; 2 School of Public Health, Xiamen University, Xiamen, Fujian, China; 3 Xiamen Guankou Hospital, Xiamen, Fujian, China

**Keywords:** adropin, community-based cohort, diabetes, diabetic kidney disease, estimated glomerular filtration rate

## Abstract

**Introduction:**

Adropin is an energy homeostasis–related peptide implicated in metabolic and vascular regulation, but its relationship with diabetic kidney disease (DKD) remains unclear. We examined cross-sectional and longitudinal associations between serum adropin and DKD and complemented the human findings with experimental validation in mice.

**Research design and methods:**

Participants were drawn from a community-based cohort in Guankou Town, Xiamen, China (2013–2021). The index visit was defined as each participant’s first health examination during 2013–2015. Cross-sectional associations with prevalent DKD were evaluated using multivariable logistic regression and restricted cubic spline (RCS) models. Longitudinal associations with incident DKD were assessed using Cox proportional hazards models and RCS. An STZ-induced DKD mouse model was used to assess adropin dynamics during disease progression and the effects of adropin administration.

**Results:**

Among 6,896 participants, higher serum adropin was inversely associated with prevalent DKD (adjusted OR per 1-unit increase 0.83 [95% CI 0.71–0.96]). Among participants free of DKD at baseline, 749 incident DKD cases occurred over a median follow-up of 6.09 years; higher baseline adropin was associated with lower incident DKD risk [adjusted HR per 1-unit increase 0.88 (0.85–0.91)]. RCS analyses suggested a largely linear cross-sectional association and a non-linear prospective association. In mice, serum adropin declined during DKD progression, and adropin treatment improved renal injury–related indicators compared with untreated DKD controls.

**Conclusion:**

Serum adropin was inversely associated with both prevalent and incident DKD in a community-based cohort, and experimental findings support a potential renoprotective role of adropin.

## Introduction

Diabetic kidney disease (DKD) is a prevalent and clinically consequential complication of diabetes mellitus, representing a major contributor to the global burden of chronic kidney disease (CKD) and cardiovascular morbidity and mortality (1–3). Global analyses indicate that CKD attributable to type 2 diabetes increased markedly between 1990 and 2021, with particularly pronounced growth among adults aged 60–74 years, underscoring a sustained public health challenge (4). Community-based evidence further suggests that DKD is common outside tertiary care settings; for instance, a large cross-sectional survey among rural residents in China reported a DKD prevalence of approximately 2.9% (5). The substantial disease burden and healthcare utilization associated with DKD highlight an urgent need to improve population-oriented risk assessment.

DKD is clinically heterogeneous, typically manifesting as albuminuria and/or progressive decline in glomerular filtration rate (GFR), with disease trajectories shaped by interrelated metabolic and hemodynamic perturbations (1, 2, 6). Identifying pathway that reflect upstream metabolic and vascular stress may therefore be particularly valuable for understanding the risk of reduced kidney function among individuals with diabetes. In this context, adropin—a secreted peptide encoded by the energy homeostasis-associated gene (ENHO)—has emerged as a potential pathway linking metabolic regulation with vascular biology. Beyond its initial description as a factor associated with energy homeostasis (7) and substrate utilization, adropin has been implicated in pathways directly relevant to DKD pathogenesis, including insulin sensitivity and glucose utilization (8, 9), endothelial function and microvascular homeostasis (10–12), and attenuation of oxidative stress and inflammation (12, 13). Because circulating adropin levels vary with metabolic characteristics and cardiometabolic risk profiles (14–17), adropin may capture upstream dysmetabolism and vascular stress that precede overt renal dysfunction.

Accumulating evidence supports a potential link between adropin and diabetic kidney injury from both human and experimental studies. Lower circulating adropin has been reported in patients with type 2 diabetes and early renal damage or diabetic nephropathy phenotypes (18–20), and emerging data suggest a possible association with CKD/DKD progression (21). Mechanistically, animal studies have demonstrated renal adropin immunoreactivity and suggested that adropin-based interventions may attenuate renal lipid toxicity, oxidative stress injury, and mitochondrial dysfunction (22). However, existing human evidence is still largely derived from cross-sectional analyses and selected clinical samples, limiting generalizability and constraining inference regarding temporality and incident risk (18–20). In addition, few studies have integrated population-based epidemiologic evidence with experimental intervention to strengthen biological interpretation.

From an epidemiologic standpoint, evaluating adropin in both cross-sectional and prospective frameworks within the same population can strengthen interpretation by jointly addressing prevalence patterns and subsequent incident risk. This is particularly relevant for DKD, where risk is multifactorial and shaped by established determinants such as hyperglycemia, hypertension, dyslipidemia, and smoking (23–26). However, longitudinal, community-based evidence examining baseline adropin in relation to incident DKD, alongside cross-sectional comparisons at baseline, remains limited.

Therefore, using a community-based dynamic cohort with follow-up from 2013 to 2021, we aimed to: (1) examine cross-sectional associations between baseline serum adropin and prevalent DKD at the index visit; (2) evaluate prospective associations between baseline adropin and incident DKD during follow-up; and (3) provide complementary experimental evidence by characterizing adropin dynamics during DKD progression and assessing the effects of adropin administration in an STZ-induced DKD mouse model. By combining cross-sectional and longitudinal analyses in the same community cohort and triangulating these findings with experimental intervention, this study seeks to clarify the clinical relevance of adropin in DKD, particularly within the reduced-eGFR phenotype captured in health-examination settings.

## Methods

### Study population and design

**Figure 1:**
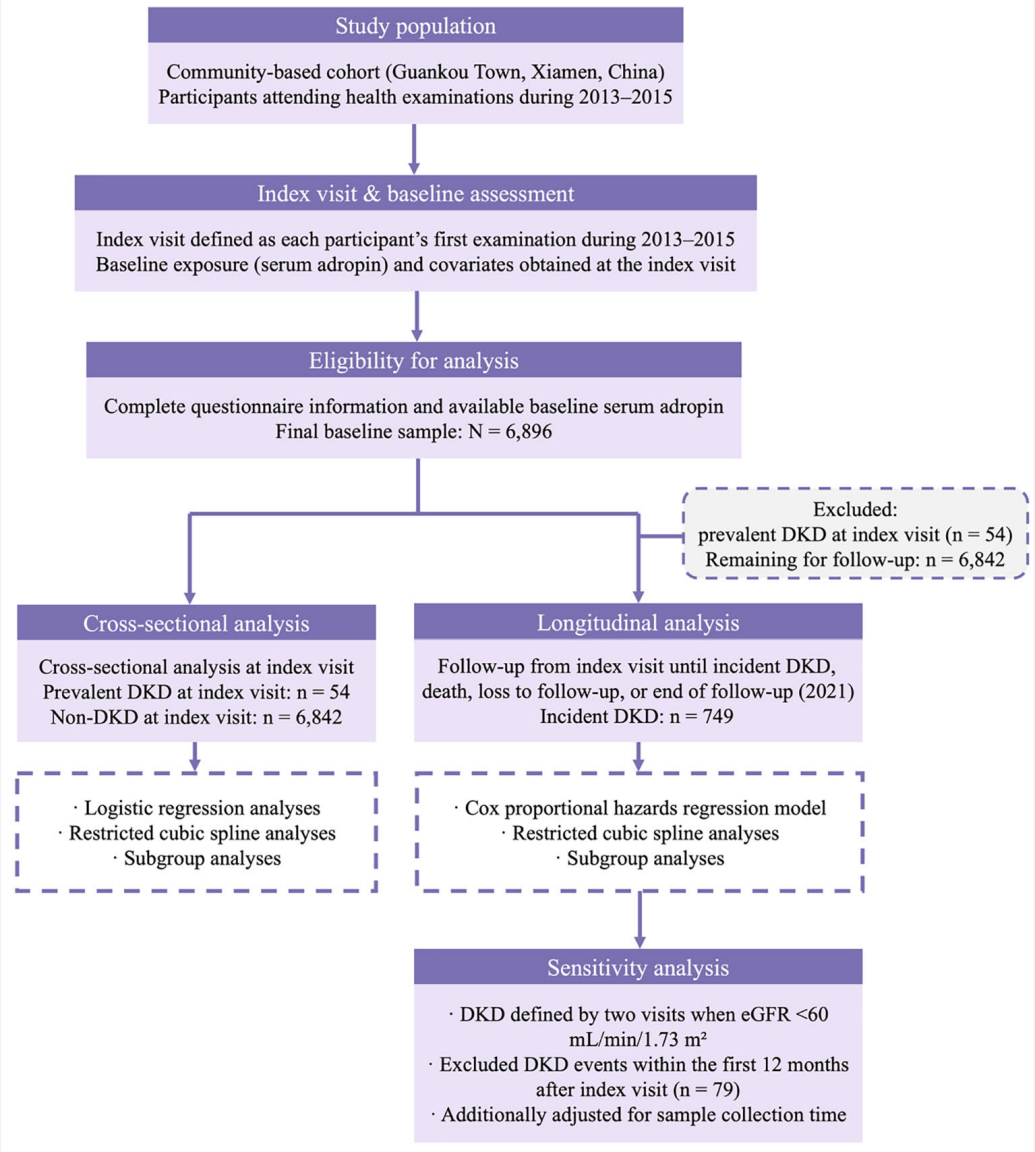
Flowchart of study design and participant selection. Eligible participants were included for cross-sectional analyses to assess the association between serum adropin levels and prevalent DKD at the index visit. For the longitudinal analysis, participants with prevalent DKD at baseline were excluded, and incident DKD events after the index visit were recorded during follow-up.

Participants were recruited from a community-based prospective cohort in Guankou Town, Xiamen, China, established to investigate chronic disease outcomes with follow-up from 2013 to 2021. Because some participants attended health examinations more than once, the index visit was defined as each participant’s first health examination conducted during 2013–2015; baseline serum samples and covariates were obtained at the index visit (**Figure 1**).

For the cross-sectional analysis, participants were included if serum adropin and prespecified covariates were available at the index visit. For the longitudinal analysis of incident DKD, participants with prevalent DKD at the index visit were excluded. Participants were followed from the index visit until the first occurrence of DKD, death, loss to follow-up, or the end of follow-up (2021), whichever came first.

The study protocol was approved by the Medical Ethics Committee of Xiamen University (Approval No. XDYX202208H04), and written informed consent was obtained from all participants.

### Data collection and laboratory measurements

At baseline and annual follow-up visits, information on residential address, demographic characteristics, lifestyle factors, comorbidities, and medication use were collected using a structured questionnaire. Physical examination data, including anthropometric measurements and laboratory tests, were obtained through an electronic medical record system.

Fasting venous blood samples were collected at the index visit, processed according to a standardized protocol, and stored at −80 °C. Serum adropin concentrations were measured using a commercially available ELISA kit. All samples were analyzed in duplicate. The intra-assay and inter-assay coefficients of variation were 2.3% and 4.1%, respectively.

### Outcome and covariates

In this study, DKD was operationally defined as the coexistence of diabetes mellitus and the reduced kidney function. Diabetes mellitus was defined as fasting plasma glucose (FPG) ≥7.0 mmol/L and/or current use of antidiabetic medications. Reduced kidney function was defined as eGFR <60 mL/min/1.73 m²; self-reported CKD/renal disease was collected as supportive information. Albuminuria measures (e.g., UACR) were not available; therefore, DKD was operationally defined as diabetes mellitus plus reduced eGFR (<60 mL/min/1.73 m²), capturing a clinically relevant reduced-eGFR diabetic CKD phenotype that is readily identifiable in health-examination and administrative data settings and is consistent with guideline frameworks in which diabetes-related kidney disease/CKD is defined by reduced eGFR and/or albuminuria.

eGFR was calculated using the 2009 Chronic Kidney Disease Epidemiology Collaboration (CKD-EPI) equation: (1) females with serum creatinine ≤ 0.7 mg/dL: GFR = 144 ×(Scr/0.7)^-0.329^ ×(0.993)^age^; (2) females with serum creatinine > 0.7 mg/dL: GFR = 144 ×(Scr/0.7)^-1.209^ ×(0.993)^age^; (3) males with serum creatinine ≤ 0.9 mg/dL: GFR = 141 ×(Scr/0.9)^-0.411^ ×(0.993)^age^; (4) males with serum creatinine > 0.9 mg/dL: GFR = 141 ×(Scr/0.9)^-1.209^ ×(0.993)^age^.

For cross-sectional analyses, prevalent DKD was assessed at the index visit using the operational definition (diabetes mellitus plus eGFR <60 mL/min/1.73 m²). For longitudinal analyses, incident DKD was ascertained at the first follow-up visit at which the operational criteria were met, and the event date was assigned as the date of that first qualifying visit. In sensitivity analyses, a stricter confirmation-based definition was additionally applied, requiring eGFR <60 mL/min/1.73 m² at two consecutive visits. This strategy was prespecified to strengthen outcome ascertainment while retaining the advantages of a health-examination cohort. Using the first qualifying visit as the event date provides a consistent and timely definition of onset for time-to-event modeling and maximizes the number of observable events, thereby improving precision. At the same time, because CKD is clinically defined by persistence over time, we repeated analyses with a confirmation-based definition requiring repeat eGFR <60 mL/min/1.73 m². This stricter definition improves specificity and better approximates CKD chronicity within the constraints of annual examination data, allowing us to demonstrate that the main findings are robust to a more stringent outcome definition.

Covariates assessed at the index visit included age, sex, body mass index (BMI), smoking status, alcohol consumption, hypertension status, and medication use.

### Statistical analyses

Continuous variables were summarized as mean ± standard deviation (SD) or median (interquartile range) and compared using Student’s t-test or the Mann–Whitney U test, as appropriate. Categorical variables were compared using the chi-square test. Missing values of the variables of interest were imputed using multiple imputation. Missing data for variables of interest were handled using multiple imputation, which was specified with m = 5 imputed datasets, maxit = 10 iterations and PMM method.

For the cross-sectional analysis, univariable and multivariable logistic regression models were used to estimate odds ratios (ORs) and 95% confidence intervals (CIs) for prevalent DKD in relation to serum adropin (continuous and quartiles), with trend tests across quartiles. Restricted cubic spline (RCS) models were used to examine potential non-linear dose–response relationships, with three knots specified.

For the longitudinal analysis, univariable and multivariable Cox proportional hazards models were used to estimate hazard ratios (HRs) and 95% CIs for incident DKD in relation to serum adropin (continuous and quartiles), with trend tests across quartiles and RCS analyses, with three knots specified. The proportional hazards assumption was assessed using Schoenfeld residuals based on the *survminer* package in R. Both graphical inspection of the scaled Schoenfeld residual plots and statistical tests were performed. A two-sided P value > 0.05, or with an approximately horizontal trend of the smoothed residual curve, was considered to indicate no violation of the proportional hazard assumption.

Subgroup analyses were conducted in both cross-sectional and longitudinal settings stratified by sex (male/female) and age (≤60 years and >60 years).

All analyses were conducted using R software. Statistical tests were two-sided, and P <0.05 was considered statistically significant.

### Sensitivity analyses

First, to improve outcome specificity and better approximate CKD chronicity within annual examination data, the association analyses were repeated using a confirmation-based definition in which reduced kidney function was considered confirmed when eGFR <60 mL/min/1.73 m² was observed at two consecutive visits. Under this definition, incident DKD was defined as diabetes mellitus plus confirmed reduced kidney function, and the event date was assigned to the second qualifying visit.

Second, to reduce potential reverse causation from subclinical disease at baseline, Cox models were repeated after excluding participants who developed DKD within one year after the index visit.

Third, to account for potential confounding related to calendar time and sample processing, analyses were further repeated with additional adjustment for sample collection time (index-visit year).

### Animal experiment

**Figure 2:**
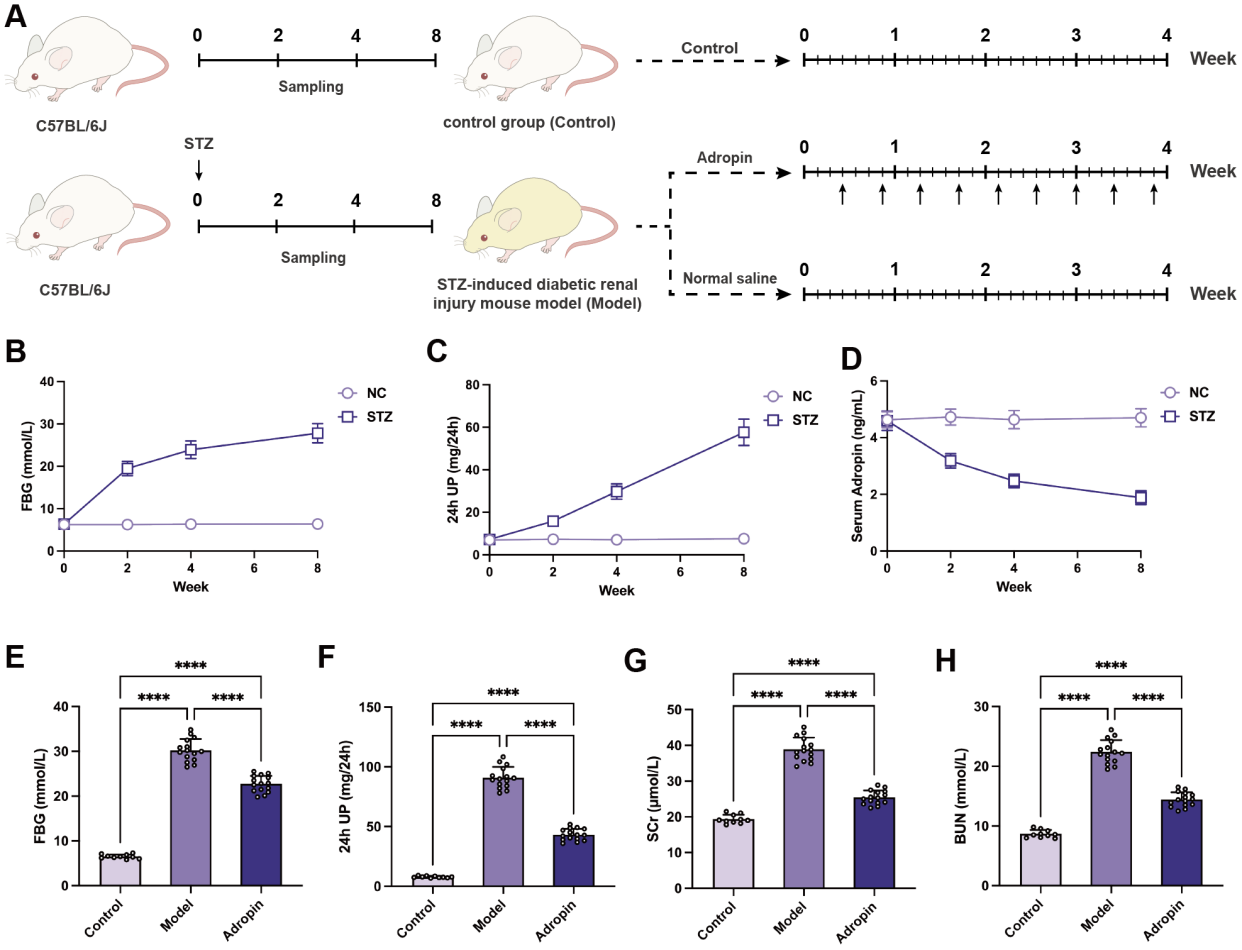
Effects of adropin intervention in an STZ-induced mouse model of diabetic kidney disease. **(A)** Schematic overview of the experimental design. **(B–D)** Fasting blood glucose (FBG), 24-h urinary protein excretion, and serum adropin levels measured at 8 weeks after DKD induction (prior to intervention). **(E–H)** FBG, 24-h urinary protein excretion, serum creatinine (Scr), and blood urea nitrogen (BUN) measured after 4 weeks of treatment in DKD mice receiving adropin or vehicle. ****p < 0.0001. One-way ANOVA was used for multiple comparisons.

A total of 40 male mice were included in the first phase to investigate changes in adropin during DKD progression. Mice were randomly assigned to a normal control group (Control, n = 10) or a STZ-induced diabetic renal injury mouse model (Model n = 30) (27, 28). Diabetes was induced by a single intraperitoneal injection of STZ (150 mg/kg) dissolved in citrate buffer, whereas control mice received an equal volume of citrate buffer alone (**Figure 2A**). One week after STZ administration, fasting blood glucose was measured, and mice with blood glucose >11.1 mmol/L were considered diabetic. Thereafter, 24-h urine was collected weekly. STZ-induced diabetic renal injury mouse model was considered established when hyperglycemia was present and 24-h urinary total protein excretion remained elevated for two consecutive weeks.

For the intervention experiment, 30 model mice were randomly assigned to a model group (n = 15) or an adropin treatment group (Adropin group, n = 15). Mice in the adropin group received adropin via tail-vein injection at a dose of 450 nmol/kg (9, 29), while mice in the model group received an equivalent volume of normal saline. Treatments were administered once every three days for four weeks. During the intervention period, fasting blood glucose, 24-h urinary total protein excretion, serum creatinine, and blood urea nitrogen were measured weekly, and serum adropin levels were assessed at the end of the intervention. All procedures were conducted in accordance with the guidelines and regulations of the Experimental Animal Administration Committee of Xiamen University, and efforts were made to minimize animal suffering throughout the study.

## Results

### Baseline characteristics

The study included 6,896 participants, with a mean age of 65.09 ± 9.47 years. At baseline, 54 participants (0.78%) were identified with prevalent DKD. Compared with the non-DKD group, participants with DKD had significantly lower serum adropin levels (4.62 ± 1.89 vs. 5.31 ± 1.90 ng/mL, P = 0.009). The DKD group also exhibited higher body weight, waist circumference, and fasting plasma glucose levels, as well as a higher prevalence of diabetes. There were no significant differences in sex distribution, smoking status, alcohol consumption, or hypertension prevalence between the two groups (**Table 1**).

**Table 1:** Baseline characteristics of participants according to DKD status at the index visit.

Variable	Non-DKD	DKD	Overall	p value
n	6842	54	6896	
adropin (ng/mL)	5.31 (1.90)	4.62 (1.89)	5.30 (1.90)	0.009
Sex (%)				0.619
Male	2827 (41.3)	20 (37.0)	2847 (41.3)	
Female	4015 (58.7)	34 (63.0)	4049 (58.7)	
age (years)	65.11 (9.45)	62.52 (10.70)	65.09 (9.47)	0.045
weight (kg)	58.21 (10.12)	64.21 (10.73)	58.25 (10.14)	<0.001
pulse (bpm)	78.60 (9.12)	78.41 (8.00)	78.60 (9.11)	0.879
BMI (kg/m^2^)	23.16 (3.29)	24.02 (3.07)	23.17 (3.29)	0.056
WC (cm)	82.43 (9.52)	86.81 (9.30)	82.47 (9.53)	0.001
FPG (mmol/L)	5.32 (1.56)	9.39 (3.32)	5.35 (1.62)	<0.001
Smoke (%)				1.000
Yes	1165 (17.0)	9 (16.7)	1174 (17.0)	
No	5677 (83.0)	45 (83.3)	5722 (83.0)	
Alcohol (%)				0.406
Yes	823 (12.0)	4 (7.4)	827 (12.0)	
No	6019 (88.0)	50 (92.6)	6069 (88.0)	
Hypertension (%)				1.000
Yes	2997 (43.8)	24 (44.4)	3021 (43.8)	
No	3845 (56.2)	30 (55.6)	3875 (56.2)	
Diabetes (%)				<0.001
Yes	573 (8.4)	54 (100.0)	627 (9.1)	
No	6269 (91.6)	0 (0.0)	6269 (90.9)	
CKD (%)				<0.001
Yes	434 (6.3)	54 (100.0)	488 (7.1)	
No	6408 (93.7)	0 (0.0)	6408 (92.9)	

BMI, Body Mass Index; WC, Waist circumference; FPG, Fasting plasma glucose; CKD, Chronic Kidney Disease.

### Cross-sectional association between adropin and prevalent DKD

Multivariable logistic regression analyses showed that higher serum adropin levels were associated with lower odds of prevalent DKD. After full adjustment, each 1-unit increase in adropin was associated with 17% lower odds of DKD (AOR = 0.83, 95% CI: 0.71-0.96; P = 1.53×10^-2^) (**Table 2**; **Supplementary Figure 1A**).

**Table 2:** Multivariable logistic regression and multivariable cox proportional hazards regression analyses of the association between serum adropin and prevalent/incident DKD.

Method/Level	Model 1		Model 2		Model 3	
	OR/HR (95% CI)	P-value	OR/HR (95% CI)	P-value	OR/HR (95% CI)	P-value
Logistic						
adropin	0.82 (0.70,0.95)	8.84×10^-3^	0.82 (0.70, 0.96)	1.26×10^-2^	0.83(0.71,0.96)	1.53×10^-2^
Q1	Ref.		Ref.		Ref.	
Q2	0.63 (0.32, 1.23)	1.85×10^-1^	0.64 (0.32, 1.24)	1.92×10^-1^	0.63 (0.31, 1.23)	1.83×10^-1^
Q3	0.50 (0.23, 1.01)	5.97×10^-2^	0.51 (0.24, 1.04)	7.27×10^-2^	0.53 (0.25, 1.08)	8.93×10^-2^
Q4	0.32 (0.12, 0.71)	8.29×10^-3^	0.34 (0.13, 0.75)	1.26×10^-2^	0.35 (0.14, 0.78)	1.52×10^-2^
P trend	4.13×10^-3^		6.82×10^-3^		9.07×10^-3^	
Cox						
adropin	0.88 (0.85, 0.91)	9.69×10^-11^	0.88 (0.85, 0.91)	9.61×10^-11^	0.88 (0.85, 0.91)	8.40×10^-11^
Q1	Ref.		Ref.		Ref.	
Q2	0.84 (0.70, 1.02)	7.39×10^-2^	0.84 (0.70, 1.02)	6.91×10^-2^	0.84 (0.70, 1.01)	6.67×10^-2^
Q3	0.73 (0.60, 0.88)	1.09×10^-3^	0.75 (0.62, 0.91)	3.18×10^-3^	0.75 (0.62, 0.91)	3.34×10^-3^
Q4	0.46 (0.37, 0.57)	3.37×10^-12^	0.45 (0.36, 0.57)	2.77×10^-12^	0.45 (0.36, 0.56)	2.42×10^-12^
P trend	1.03×10^-12^		1.83×10^-12^		1.73×10^-12^	

a Model 1 was an unadjusted model including only Adropin.

b Model 2 was adjusted for age, sex, body mass index (BMI), smoking status, and alcohol consumption.

c Model 3 was further adjusted for hypertension and medication use based on Model 2.

**Figure 3:**
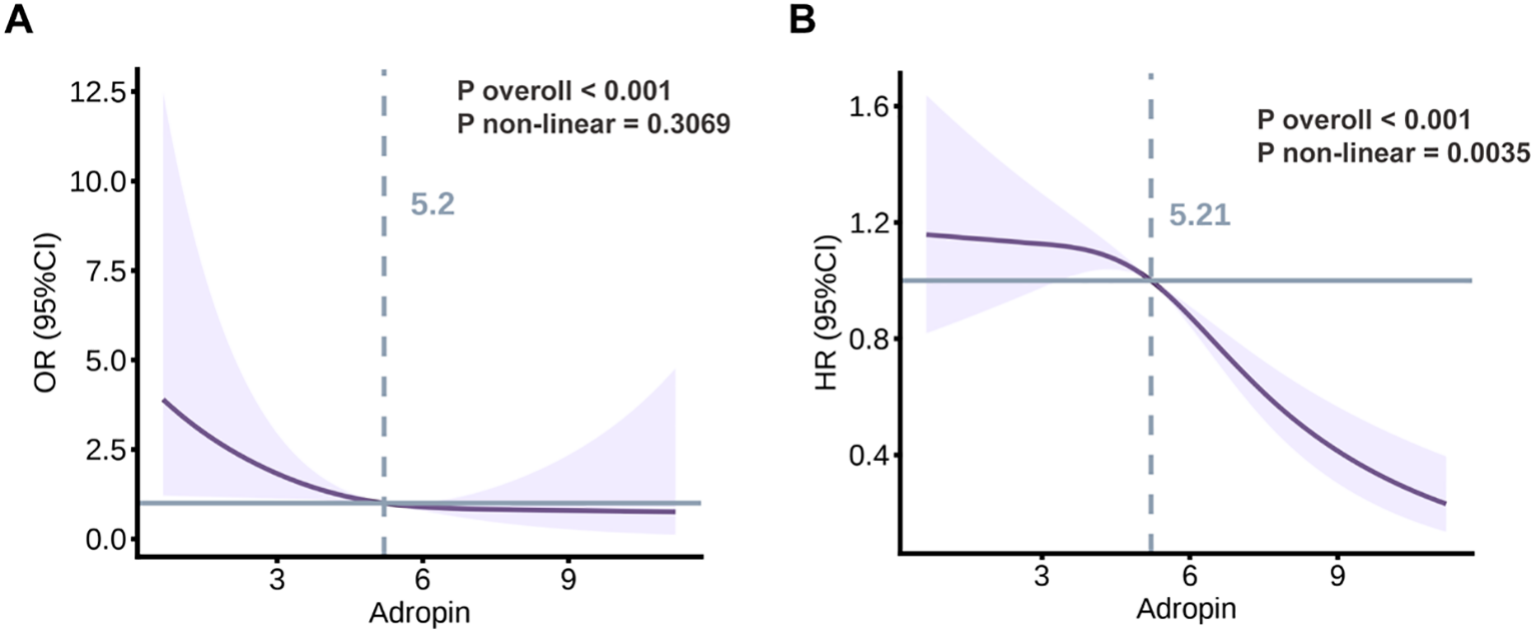
RCS analyses of the association between serum adropin and DKD. **(A)** RCS analysis of the cross-sectional dose–response association between serum adropin and prevalent DKD. **(B)** RCS analysis of the longitudinal dose–response association between serum adropin and incident DKD. Solid lines represent adjusted estimates and shaded areas indicate 95% CIs. The vertical dashed line denotes the reference value of serum adropin. Models were adjusted for age, sex, BMI, smoking status, alcohol consumption, hypertension and medication use.

When adropin was categorized into quartiles (Q1 ≤ 3.94 ng/mL; 3.94 < Q2 ≤ 5.20; 5.20 < Q3 ≤ 6.55; Q4 > 6.55), participants in the highest quartile (Q4) had significantly lower odds of DKD than those in Q1 across all models, and a significant trend was observed with increasing adropin levels (P for trend < 0.01) (**Table 2**; **Supplementary Figure 1A**). RCS analyses suggested a linear inverse dose–response relationship between adropin and prevalent DKD (P for overall association < 0.001; P for nonlinearity = 0.3069) (**Figure 3A**).

Subgroup analyses indicated that the inverse association was significant among male participants and those aged ≤60 years, whereas no significant association was observed in female participants or those aged >60 years (**Table 3**; **Supplementary Figure 2A**).

**Table 3:** Subgroup analyses of the association between serum adropin and DKD.

Method/Group	Model 1		Model 2		Model 3	
	OR/HR (95% CI)	P-value	OR/HR (95% CI)	P-value	OR/HR (95% CI)	P-value
Logistic						
Sex						
male	0.74 (0.57, 0.95)	0.018	0.75 (0.58, 0.97)	0.026	0.74 (0.56, 0.96)	0.023
female	0.87 (0.72, 1.05)	0.146	0.87 (0.72, 1.05)	0.150	0.88 (0.73, 1.06)	0.181
Age						
≤60	0.75 (0.59, 0.94)	0.012	0.75 (0.59, 0.94)	0.012	0.74 (0.59, 0.94)	0.014
>60	0.89 (0.73, 1.09)	0.261	0.90 (0.73, 1.1)	0.294	0.90 (0.73, 1.10)	0.298
Cox						
Sex						
male	0.90 (0.84, 0.96)	0.001	0.90 (0.85, 0.96)	0.001	0.90 (0.85, 0.96)	0.001
female	0.87 (0.83, 0.91)	<0.001	0.87 (0.83, 0.91)	<0.001	0.87 (0.83, 0.91)	<0.001
Age						
≤60	0.87 (0.81, 0.93)	<0.001	0.87 (0.81, 0.93)	<0.001	0.87 (0.81, 0.93)	<0.001
>60	0.88 (0.84, 0.92)	<0.001	0.89 (0.85, 0.93)	<0.001	0.89 (0.84, 0.93)	<0.001

a Model 1 was an unadjusted model including only Adropin.

b Model 2 was adjusted for age, sex, body mass index (BMI), smoking status, and alcohol consumption.

c Model 3 was further adjusted for hypertension and medication use based on Model.

### Longitudinal association between adropin and incident DKD

During a median follow-up of 6.09 years (IQR, 3.59–7.60 years), 749 incident DKD cases occurred among 6,842 participants free of DKD at baseline. In multivariable Cox models, each 1-unit increase in adropin was associated with a 12% lower risk of incident DKD (AHR = 0.88, 95% CI: 0.85–0.91; P < 0.001) (**Table 2**; **Supplementary Figure 1B**).

In quartile analyses, participants in Q3 and Q4 had significantly lower hazards of incident DKD compared with Q1, and a strong dose–response trend was observed (P for trend < 0.001) (**Table 2**; **Supplementary Figure 1B**).

Unlike the cross-sectional findings, RCS analysis in the longitudinal setting indicated a non-linear association (*P* for nonlinearity = 0.0035), suggesting that the protective effect may be more pronounced at higher adropin concentrations (**Figure 3B**).

In subgroup analyses, higher adropin levels remained significantly associated with lower incident DKD risk across sex and age strata (**Table 3**; **Supplementary Figure 2B**).

### Sensitivity analyses

First, to address potential outcome misclassification based on a single low eGFR value, we repeated the Cox proportional hazards analyses using a stricter, confirmation-based definition of incident DKD. Among participants who met the incident DKD definition in the main analysis, 287 cases satisfied this confirmation requirement. Using this stricter definition, the inverse association between serum adropin and incident DKD remained consistent (per 1-unit increase: HR = 0.85, 95% CI 0.80–91; P = 4.25×10^-7^), and quartile-based analyses yielded similar results (Q4 vs Q1: HR = 0.33, 95% CI 0.23–0.47; P for trend = 1.44×10^-9^) (**Supplementary Table 1**).

Second, to reduce potential reverse causation from subclinical disease at baseline, we excluded participants who developed DKD within 1 year after baseline and refitted the Cox models. The associations were materially unchanged (per 1-unit increase: HR = 0.88, 95% CI 0.85–0.91; P = 1.76×10^-^¹^0^ in the fully adjusted model), and quartile-based analyses were consistent (Q4 vs Q1: HR = 0.46, 95% CI 0.36–0.57; P for trend = 6.86×10^-^¹²) (**Supplementary Table 2**).

Third, after additional adjustment for sample collection time, the inverse association between adropin and incident DKD remained stable (per 1-unit increase: HR = 0.88, 95% CI 0.85–0.92; P = 1.90×10^-^¹^0^; Q4 vs Q1: HR = 0.46, 95% CI 0.36–0.57; P for trend = 7.37×10^-^¹²) (**Supplementary Table 3**).

### Experimental validation in mice

In the observational phase, compared with the normal control (Control) group, STZ-treated mice developed a progressive diabetic phenotype characterized by sustained hyperglycemia and worsening renal injury. Fasting blood glucose increased after STZ administration and remained markedly elevated throughout the 8-week DKD induction period (**Figure 2B**). In parallel, 24-h urinary total protein excretion increased over time, indicating progressive proteinuria (**Figure 2C**). Notably, serum adropin levels decreased during DKD progression in STZ-treated mice relative to controls (**Figure 2D**), suggesting that declining circulating adropin accompanies the development of diabetic renal injury in this model.

In the intervention phase, model mice were treated with adropin or vehicle for 4 weeks. Compared with vehicle-treated model mice, adropin administration led to consistent improvements in metabolic and renal injury indicators. Specifically, fasting blood glucose and 24-h urinary total protein excretion were significantly reduced in the adropin-treated group during the intervention period (**Figures 2E, F**). Markers of renal functional impairment were also improved: serum creatinine and blood urea nitrogen were lower in adropin-treated mice than in vehicle-treated model mice (**Figures 2G, H**). Although these parameters improved, they generally did not fully normalize to Control levels, indicating partial but meaningful attenuation of DKD-related injury (**Figures 2E–H**).

## Discussion

In this study, we integrated population-based epidemiologic analyses with experimental validation to evaluate the role of serum adropin in diabetic kidney disease (DKD). Using a community-based dynamic cohort with up to 6–7 years of follow-up, lower adropin levels were consistently associated with both prevalent and incident DKD. In parallel, adropin administration attenuated renal injury indicators in an STZ-induced DKD mouse model. Together, these findings provide a coherent translational evidence chain linking lower adropin to higher DKD risk and kidney injury.

### Association between adropin and DKD

In the cross-sectional analysis, serum adropin levels were inversely associated with prevalent DKD, and dose–response modeling suggested a linear relationship. These results are broadly consistent with previous clinical studies reporting lower circulating adropin levels in patients with DKD/diabetic nephropathy than in controls (19, 30), including studies focused on early renal damage in type 2 diabetes (20). Adropin is implicated in endothelial regulation and energy homeostasis, and reduced adropin levels have been linked to insulin resistance, endothelial dysfunction, and systemic inflammation (8, 10, 13, 16, 31, 32)— mechanistic pathways highly relevant to DKD pathogenesis. Meanwhile, the slightly younger age observed among participants with prevalent DKD should be interpreted cautiously given the small number of baseline cases. In community-based populations, earlier onset of renal impairment among some younger diabetic individuals may reflect heterogeneity in metabolic control, susceptibility, or survivor bias rather than a protective effect of older age.

Although the direction of association has been broadly consistent, reported effect sizes vary across studies. For example, Hu et al. (18) reported a stronger inverse association in a clinic-based population using logistic regression. Such heterogeneity is expected given differences in sampling frames and disease spectrum: clinic-based case–control studies often enrich for more advanced disease and sharper contrasts, whereas our cohort, drawn from routine health examinations, captures a broader risk spectrum closer to real-world population risk. In addition, because albuminuria was unavailable, early albuminuric DKD with preserved eGFR may have been under-ascertained at baseline, which would lower the observed prevalence and could attenuate cross-sectional contrasts. Notably, beyond cross-sectional comparisons, our study assessed both prevalent and incident DKD, and the inverse association was prospectively replicated using time-to-event analyses, strengthening temporal interpretation.

In the longitudinal analysis, higher baseline adropin was associated with a lower risk of incident DKD. Restricted cubic spline analyses suggested a non-linear prospective dose–response relationship, with greater risk separation at higher adropin concentrations, compatible with a threshold- or saturation-like pattern. Replication in independent cohorts will be important to confirm the curve shape and to determine whether any inflection point is clinically meaningful. Consistent with this prognostic interpretation, a 2025 study in patients with type 2 diabetes reported that serum adropin was associated with CKD progression defined by eGFR decline (21). Because albuminuria measures were unavailable, our outcome should be interpreted as diabetes with reduced kidney function, i.e., a reduced-eGFR diabetic CKD phenotype that may include non-albuminuric DKD. Recent evidence suggests that non-albuminuric DKD represents an important and increasingly recognized phenotype of diabetic kidney disease, characterized by progressive decline in kidney function despite the absence of significant albuminuria (33, 34).This phenotype is clinically important, particularly in community screening contexts where eGFR is routinely measured, and it captures individuals at high risk of adverse renal and cardiovascular outcomes.

To strengthen outcome validity under this data structure, we conducted a confirmation-based sensitivity analysis requiring eGFR <60 mL/min/1.73 m² at two consecutive visits, which improves specificity and better approximates chronic kidney dysfunction within annual examination data. The inverse association between adropin and incident DKD remained consistent under this stricter definition, supporting that the primary finding is not driven solely by transient low eGFR values and strengthening confidence that lower adropin is robustly associated with incident reduced-eGFR kidney disease in individuals with diabetes. Additional sensitivity analyses excluding early events and adjusting for sample collection time produced materially similar estimates, further reducing concerns about reverse causation and calendar-time–related confounding.

### Experimental evidence of renoprotection

To complement observational findings and strengthen biological inference, we conducted both a natural-history and an intervention experiment in an STZ-induced diabetic renal injury mouse model. The criterion of sustained proteinuria for two consecutive weeks was used as an experimental indicator of diabetic renal injury rather than a direct equivalent of the clinical diagnostic criteria for DKD. Given the accelerated disease progression in rodent models, shorter observation periods are commonly adopted for model establishment studies (35).

Although the STZ-induced model predominantly reflects insulin-deficient diabetes, many downstream mechanisms of kidney injury are shared between type 1 and type 2 DKD, including hyperglycemia-driven oxidative stress, mitochondrial dysfunction, inflammatory activation, endothelial injury, and tubular damage (36, 37). Previous studies have demonstrated that adropin modulates several of these pathways (11–13). Therefore, the protective effects observed in the STZ model may reflect actions on common pathogenic mechanisms of diabetic kidney injury.

During DKD progression, STZ-treated mice developed sustained hyperglycemia, progressive increases in 24-h urinary total protein excretion, and worsening renal function indices, while circulating adropin levels declined over time. This parallel trajectory—worsening metabolic/renal phenotypes accompanied by falling adropin—mirrors the population-based associations and supports biological coherence.

In the intervention phase, adropin administration produced improvements across multiple complementary indicators of DKD-related injury. Compared with vehicle-treated model mice, the adropin-treated group exhibited lower fasting blood glucose and reduced 24-h urinary total protein excretion, together with improved renal function markers (serum creatinine and blood urea nitrogen). Importantly, these improvements were observed longitudinally during the treatment period and across both proteinuria and renal function indices, supporting a sustained attenuation of DKD-related injury. Although parameters did not fully normalize to control levels, the consistent direction of benefit across endpoints supports a biologically meaningful renoprotective effect of adropin in this model.

These findings are concordant with DKD-focused experimental literature. For example, Yu et al. reported that adropin delivered via ROS-responsive nanocapsules reduced oxidative stress injury, attenuated renal lipid toxicity, and improved mitochondrial-related pathways in diabetic mice (22). Integrating prior mechanistic evidence with our results, a plausible framework is that adropin may mitigate DKD progression through coordinated effects on metabolic stress and kidney injury pathways—particularly oxidative stress and mitochondrial dysfunction—with downstream modulation of inflammation and lipotoxicity that are central to DKD pathogenesis.

Although serum creatinine and blood urea nitrogen were measured, direct assessment of glomerular filtration rate was not performed. Furthermore, renal histopathological analyses were not conducted; thus, structural changes in kidney tissue could not be evaluated. Consequently, the renoprotective effects of adropin were assessed primarily using functional and biochemical indicators. Future studies incorporating GFR measurements and renal histology will be important for further validating and characterizing the protective role of adropin in DKD.

The strengths of this study include the integration of a large, longitudinal community cohort with experimental validation. However, several limitations must be acknowledged. First, albuminuria was unavailable; thus, early albuminuric DKD with preserved eGFR may have been missed, and our outcome primarily represents diabetes with reduced eGFR (a reduced-eGFR/diabetic CKD phenotype). Second, as an observational study, residual confounding cannot be excluded. Third, the study population was derived from a single community in China; therefore, external validation in diverse populations is required to establish generalizability.

In conclusion, in a community-based cohort with longitudinal follow-up, lower serum adropin levels were consistently associated with both prevalent and incident diabetic kidney disease. These epidemiologic findings were complemented by experimental evidence showing that adropin administration attenuated renal injury in diabetic mice.

## Data Availability

The raw data supporting the conclusions of this article will be made available by the authors, without undue reservation.
