# Supplementary material for: Serum adropin as a potential renoprotective factor in diabetic kidney disease: evidence from a Chinese elderly cohort and an experimental mouse model
Source: Front Endocrinol (Lausanne). 2026 Jul 13;17:1893876. doi: 10.3389/fendo.2026.1893876 (PMC13402196; doi:10.3389/fendo.2026.1893876)
Supplement: Supplementary file 1 [file SupplementaryFile1.docx]

**
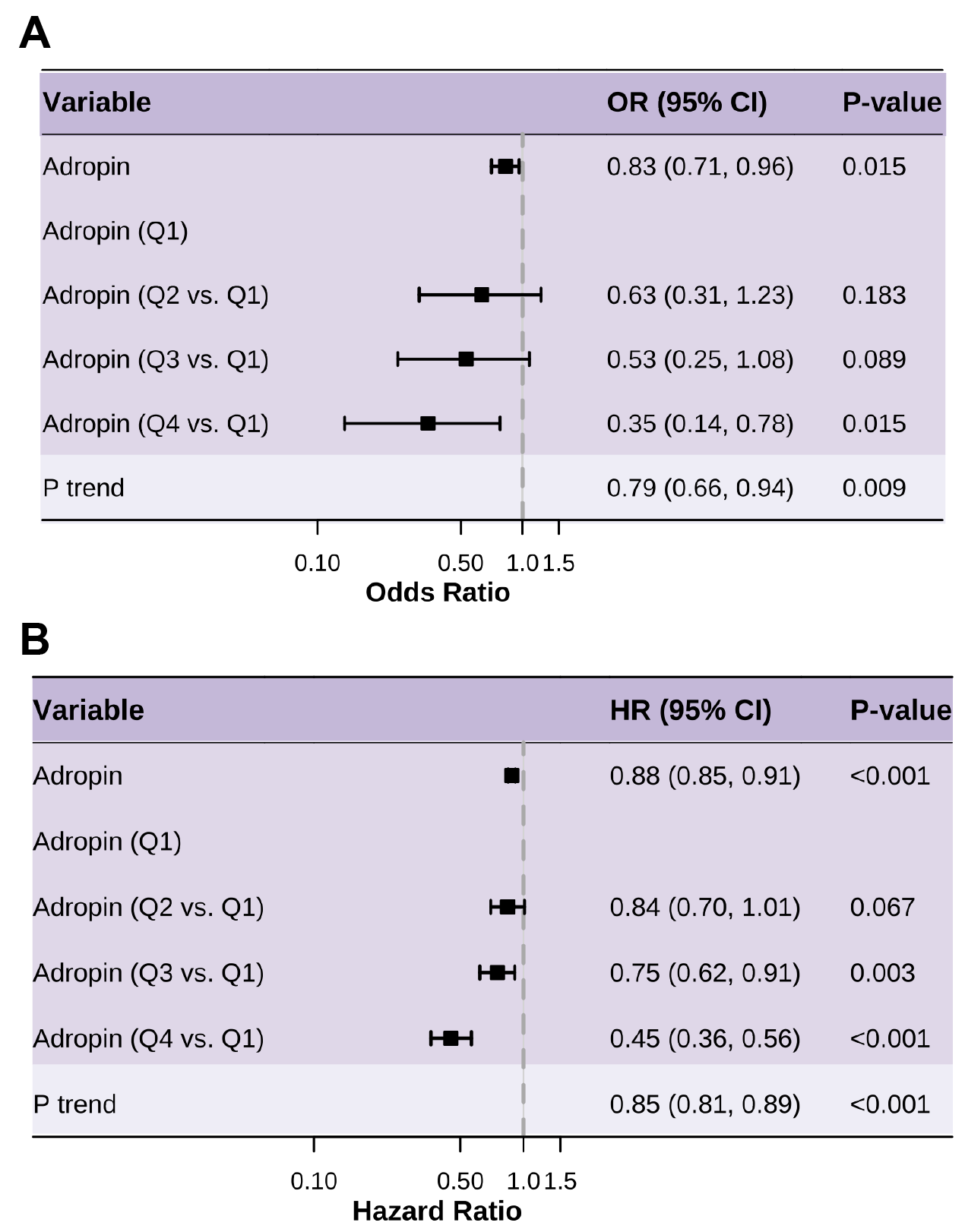
**

**Figure S1 Multivariable logistic regression and multivariable cox proportional hazards regression analyses of the association between serum adropin and prevalent/incident DKD.**

(A) Odds ratios (ORs) and 95% confidence intervals (CIs) for prevalent DKD according to serum adropin, estimated using multivariable logistic regression. (B) Hazard ratios (HRs) and 95% CIs for incident DKD according to serum adropin, estimated using multivariable Cox proportional hazards models. Models were adjusted for age, sex, BMI, smoking status, alcohol consumption, hypertension and medication use.

**
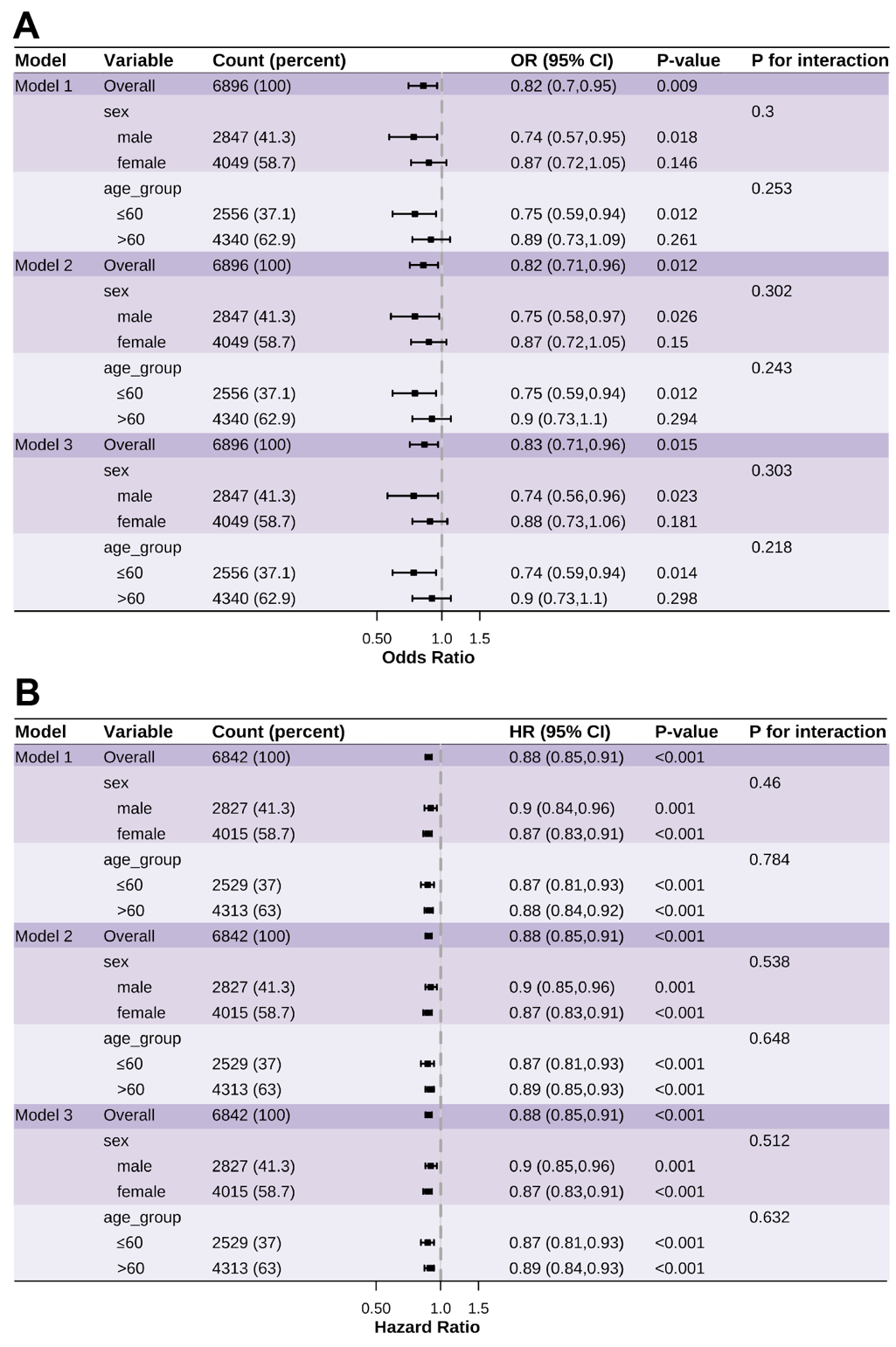
**

**Figure S2. Subgroup analyses of the association between serum adropin and DKD.**

(A) Subgroup-specific odds ratios (ORs) and 95% confidence intervals (CIs) for prevalent DKD estimated using multivariable logistic regression. (B) Subgroup-specific hazard ratios (HRs) and 95% CIs for incident DKD estimated using multivariable Cox proportional hazards models. Models were adjusted for covariates as described in the Methods.

**Table S1. Sensitivity analysis for the association between serum adropin levels and incident DKD with a confirmed decline in renal function.**

| Outcome/  Level | Model 1 | | Model 2 | | | Model 3 | |
| --- | --- | --- | --- | --- | --- | --- | --- |
|  | HR (95% CI) | P-value | HR (95% CI) | | P-value | HR (95% CI) | P-value |
| DKD | 0.85 (0.80, 0.91) | 4.95×10^-7^ | 0.85 (0.80, 0.91) | 6.88×10^-7^ | | 0.85 (0.80, 0.91) | 4.25×10^-7^ |
| Q1 | Ref. | | Ref. | | | Ref. | |
| Q2 | 0.66 (0.49, 0.89) | 6.35×10^-3^ | 0.65 (0.48, 0.88) | | 4.74×10^-3^ | 0.65 (0.48, 0.87) | 4.24×10^-3^ |
| Q3 | 0.58 (0.43, 0.79) | 4.98×10^-4^ | 0.61 (0.45, 0.83) | | 1.49×10^-3^ | 0.60 (0.44, 0.82) | 1.36×10^-3^ |
| Q4 | 0.34 (0.24, 0.49) | 6.75×10^-9^ | 0.33 (0.23, 0.48) | | 4.17×10^-9^ | 0.33 (0.23, 0.47) | 2.53×10^-9^ |
| P trend | 1.76×10^-9^ | | 2.29×10^-19^ | | | 1.44×10^-9^ | |

Reduced kidney function was confirmed when eGFR <60 mL/min/1.73 m² was observed at two consecutive visits. Incident DKD was defined as diabetes mellitus plus confirmed reduced kidney function, and association analyses were repeated using this stricter definition.

Model 1 was an unadjusted model including only adropin. Model 2 was adjusted for age, sex, body mass index (BMI), smoking status, and alcohol consumption. Model 3 was further adjusted for hypertension and medication use based on Model 2.

**Table S2. Sensitivity analysis for the association between serum adropin levels and incident DKD excluding events occurring within the first year of follow-up.**

| Outcome/  Level | Model 1 | | Model 2 | | | Model 3 | |
| --- | --- | --- | --- | --- | --- | --- | --- |
|  | HR (95% CI) | P-value | HR (95% CI) | | P-value | HR (95% CI) | P-value |
| DKD | 0.88(0.85, 0.91) | 1.90×10^-10^ | 0.88 (0.85, 0.91) | 1.54×10^-10^ | | 0.88 (0.85, 0.91) | 1.76×10^-10^ |
| Q1 | Ref. | | Ref. | | | Ref. | |
| Q2 | 0.85 (0.70, 1.02) | 8.49×10^-2^ | 0.84 (0.70, 1.02) | | 8.04×10^-2^ | 0.84 (0.70, 1.02) | 7.99×10^-2^ |
| Q3 | 0.73 (0.60, 0.89) | 1.49×10^-3^ | 0.75 (0.62, 0.91) | | 4.04×10^-3^ | 0.75 (0.62, 0.92) | 4.51×10^-3^ |
| Q4 | 0.46 (0.37, 0.58) | 1.29×10^-11^ | 0.46 (0.36, 0.57) | | 8.46×10^-12^ | 0.46 (0.36, 0.57) | 9.47×10^-12^ |
| P trend | 4.18×10^-12^ | | 5.59×10^-12^ | | | 6.86×10^-12^ | |

We excluded participants who developed DKD within one year after baseline and re-performed Cox proportional hazards analyses.

Model 1 was an unadjusted model including only adropin. Model 2 was adjusted for age, sex, body mass index (BMI), smoking status, and alcohol consumption. Model 3 was further adjusted for hypertension and medication use based on Model 2.

**Table S3. Sensitivity analysis for the association between serum adropin levels and incident DKD with exclusion of early events and adjustment for collection time.**

| Outcome/  Level | Model 1 | | Model 2 | | | Model 3 | |
| --- | --- | --- | --- | --- | --- | --- | --- |
|  | HR (95% CI) | P-value | HR (95% CI) | | P-value | HR (95% CI) | P-value |
| DKD | 0.88(0.85, 0.91) | 1.90×10^-10^ | 0.88 (0.85, 0.91) | 1.54×10^-10^ | | 0.88 (0.85, 0.92) | 1.90×10^-10^ |
| Q1 | Ref. | | Ref. | | | Ref. | |
| Q2 | 0.85 (0.70, 1.02) | 8.49×10^-2^ | 0.84 (0.70, 1.02) | | 8.04×10^-2^ | 0.85 (0.70, 1.02) | 8.16×10^-2^ |
| Q3 | 0.73 (0.60, 0.89) | 1.49×10^-3^ | 0.75 (0.62, 0.91) | | 4.04×10^-3^ | 0.75 (0.62, 0.92) | 4.61×10^-3^ |
| Q4 | 0.46 (0.37, 0.58) | 1.29×10^-11^ | 0.46 (0.36, 0.57) | | 8.46×10^-12^ | 0.46 (0.36, 0.57) | 1.02×10^-11^ |
| P trend | 4.18×10^-12^ | | 5.59×10^-12^ | | | 7.37×10^-12^ | |

We first excluded participants who developed DKD within one year after baseline as well as sample collection time was additionally adjusted as a potential confounder, and re-performed cox proportional hazards analyses.

Model 1 was an unadjusted model including only adropin. Model 2 was adjusted for age, sex, body mass index (BMI), smoking status, and alcohol consumption. Model 3 was further adjusted for hypertension, medication use and collection time based on Model 2.
